# Inter-Individual Variability of a High-Intensity Interval Training With Specific Techniques vs. Repeated Sprints Program in Sport-Related Fitness of Taekwondo Athletes

**DOI:** 10.3389/fphys.2021.766153

**Published:** 2021-11-17

**Authors:** Alex Ojeda-Aravena, Tomás Herrera-Valenzuela, Pablo Valdés-Badilla, Jorge Cancino-López, José Zapata-Bastias, José Manuel García-García

**Affiliations:** ^1^Departamento de Ciencias de la Actividad Física, Universidad de Los Lagos, Puerto Montt, Chile; ^2^Facultad de Ciencias del Deporte, Universidad de Castilla-La Mancha, Toledo, Spain; ^3^Escuela de Ciencias del Deporte y la Actividad Física, Facultad de Salud, Universidad Santo Tomás (UST), Santiago, Chile; ^4^Escuela de Ciencias de la Actividad Física, el Deporte y la Salud, Universidad de Santiago de Chile (USACH), Santiago, Chile; ^5^Departamento de Ciencias de la Actividad Física, Facultad de Ciencias de la Educación, Universidad Católica del Maule, Talca, Chile; ^6^Carrera de Entrenador Deportivo, Escuela de Educación, Universidad Viña del Mar, Viña del Mar, Chile; ^7^Exercise Science Laboratory, School of Kinesiology, Faculty of Medicine, Universidad Finis Terrae, Santiago, Chile

**Keywords:** martial arts, physical fitness, explosive strength, combat sports, physical training

## Abstract

This study investigated the effect of 4 weeks of high-intensity interval training (HIIT) with specific techniques (TS-G) vs. repeated sprints (RS-G) and analyzed the inter-individual variability [classified into responders (Rs) and non-responders (NRs)] on sport-related fitness in taekwondo (TKD) athletes. Athletes of both genders (*n* = 12) were randomly assigned into TS-G and RS-G groups. Both groups trained 3 days/week for 4 weeks [two blocks of three rounds of 2 min of activity (4-s of all-out efforts with 28-s dynamical pauses) with 1 min of recovery in between and 5 min between blocks] during their regular training. The related sport fitness assessments included squat jump (SJ), countermovement jump (CMJ), multiple frequency speed of kick test (FSKT_MULT_), specifically total kicks and Kick Decrement Index (KDI), and 20-m shuttle run (20MSR). Relevant results indicate a significant effect of the time factor in both groups for SJ performance and a significant decrease for KDI in RS-G. In addition, an improvement in performance according to the effect size analysis in the TS-G in total kicks, KDI, and 20MSR. Complementarily, a higher proportion of athlete Rs was reported in TS-G vs. RS-G for SJ (50% vs. 30.3%, respectively), CMJ, and total kicks (16.6% vs. 0%). In conclusion, the addition to the regular training of a HIIT with specific-techniques and repeated-sprints associated with intervals and similar structure of the combat during 4 weeks of training can improve the concentric characteristics of lower limb performance, although they were not the sufficient stimuli in the other components of TKD-related fitness.

## Introduction

Taekwondo (TKD) is an Olympic combat sport renowned for its fast kicks (Norjali Wazir et al., 2019), its intermittent nature (i.e., the average effort/pause ratio = 1:7 to 1:2) (da Silva Santos et al., 2018), and the high physical demand involved for the athletes (Maloney et al., 2018). For example, TKD athletes compete annually at the national and international level in at least four tournaments of four to seven combats with short recovery periods (Carazo-Vargas et al., 2015; Bridge et al., 2018). In addition, they have short rest periods between tournaments requiring them to maintain high sports fitness and to prevent the risk of injury (Kalkhoven et al., 2021). Elite TKD athletes (i.e., medalists in at least one international competition) (Norjali Wazir et al., 2019) present high general and specific physical fitness such as optimal dynamical strength performance, high cardiorespiratory fitness, and high ability to execute repeated high-intensity-specific and intermittent motor efforts (da Silva Santos and Franchini, 2018; Norjali Wazir et al., 2019).

Following these aspects, TKD coaches face several challenges in the physical preparation of TKD athletes (Reale et al., 2019). In this regard, high-intensity interval training (HIIT) has been proposed as a specific and effective modality, which, in a short time, can provide significant improvements in physical fitness related to combat sports (Franchini et al., 2019; Vasconcelos et al., 2020). In TKD, studies with the use of HIIT include protocols, consisting mostly of 3 weekly sessions added to the usual training (on alternate or double days for 4–8 weeks) based on repeated sprints (Monks et al., 2017; Seo et al., 2019; Ouergui et al., 2020, 2021). In addition, recent studies in HIIT are based on specific techniques (TS-G) (Aravena et al., 2020; Ouergui et al., 2020, 2021; Ojeda-Aravena et al., 2021). Relevant results include significant increases in aerobic fitness (Monks et al., 2017; Seo et al., 2019; Ouergui et al., 2020). Similarly, increases in specific high-intensity interval efforts include the multiple frequency speed of kick test (FSKT_MULT_) outcomes (Aravena et al., 2020) and the significant increases in jumping ability (Monks et al., 2017; Ouergui et al., 2020, 2021). However, the results among the different studies are inconsistent.

In addition to the abovementioned results, commonly, the presentation of results reported by studies is expressed in terms of group changes (i.e., mean and SD) without considering the inter-individual variability of athletes (Harriss and Atkinson, 2015; Atkinson et al., 2019; Pickering and Kiely, 2019). In this respect, this research topic has been the subject of study since the 1980s in precision medicine to find responders (Rs) and non-responders (NRs) to physical exercise treatment applied to obese sedentary individuals and/or with comorbidity and recently in the field of applied sports science to understand the responses of an athlete (Bonafiglia et al., 2016; Güllich, 2018; Ramirez-Campillo et al., 2018; Pickering and Kiely, 2019; Bratland-Sanda et al., 2020; Schulhauser et al., 2020; Talsnes et al., 2020). In this context, in combat sports and specifically in TKD, only one study is known at present (Ojeda-Aravena et al., 2021), while 163 the comparison of HIIT protocols based on TS-G vs. RS-G is 164 not yet analyzed.

Consequently, the potential efficacy of HIIT protocols with specific techniques could be an option to include during the physical preparation of athletes. In turn, an inter-individual analysis could provide useful information on the mechanisms of adaptation to training, helping coaches in training prescription. Therefore, this study investigated the effect of 4 weeks of HIIT with specific techniques vs. repeated sprints and analyzed the inter-individual variability (classified into Rs and NRs) on sport-related fitness in TKD athletes. The hypothesis is that the HIIT with specific techniques would be statistically superior to the HIIT with repeated-sprints on sport-related fitness. The rationale for the hypothesis is based on the notion that the ecological specificity of HIIT with specific techniques (i.e., the specific temporal structure of the modality, similar to that combat) could develop greater adaptations than the HIIT based on repeated-sprints.

## Materials and Methods

### Participants

**FIGURE 1:**
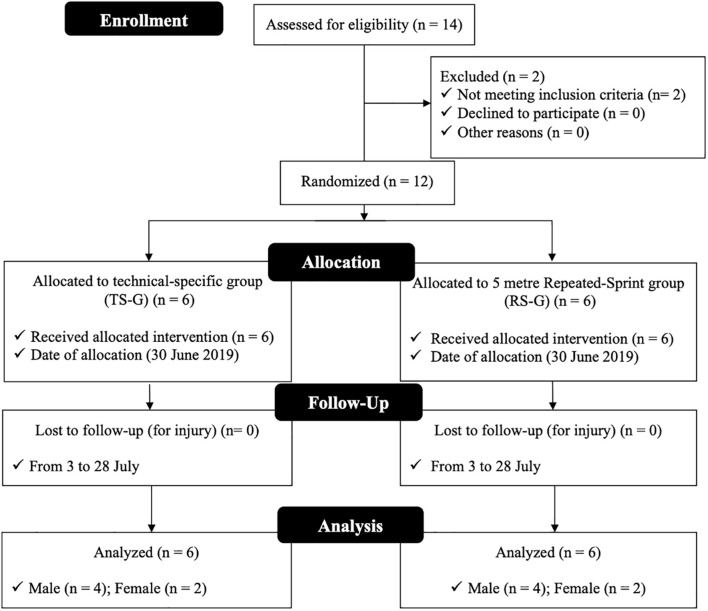
Flowchart of the process followed in the study.

A total of 12 TKD athletes of both genders distributed in females [*n* = 4, age: 16.8 ± 2.5 years, height: 155 ± 4 cm, body mass (BM): 54 ± 5 kg, percentage fat mass: 30.1 ± 5.7%, and experience: 7.4 ± 3.5 years] and males (*n* = 8, age: 17.8 ± 3.8 years, height: 165 ± 11 cm, BM: 63 ± 14 kg, percentage fat mass: 16.5 ± 4.7%, and experience: 7.4 ± 3.5 years), who compete annually in national and international level tournaments, completed this study. Athletes were invited to participate in this study during the annual planning transition period (July 2019) and randomly assigned into technical-specific group (TS-G) (*n* = 6) and repeated-sprint group (RS-G) (*n* = 6). Each group consisted of four males and two females (Figure 1). To participate, all athletes had to meet the following inclusion criteria: (i) 4 or more years of experience competing in TKD; (ii) training three or more times per week; (iii) be preparing for competitions or tournaments organized by the Federación Deportiva Nacional de Taekwondo, an organization recognized by World Taekwondo; (iv) be free of injuries and neuromuscular problems; and (vi) not be in a period of BM reduction. All athletes and/or family members of athletes under 18 years of age were informed in advance of the study purposes, associated benefits, experimental procedures, and potential risks by informed consent or informed assent before the assessments and training sessions. This study was conducted in compliance with the ethical standards for sports science studies (Harriss and Atkinson, 2015) and implemented after the approval by the Ethics Committee of the University “blind for research purposes” following the Declaration of Helsinki for work with humans (General Assembly of the World Medical Association, 2014).

### Assessments

#### Anthropometric Assessments

Height (cm) was assessed with a stadiometer (Bodymeter 206, Seca, Germany, accuracy 1 mm) following the standard protocols (An et al., 2019). BM and percentage of fat mass were assessed using an electric bioimpedance scale (InBody120, tetrapolar tactile electrode system, model BPM040S12F07, Biospace, Inc., Seoul, South Korea, accuracy to 0.1 kg) (Miller et al., 2016). The researchers administered and monitored the procedures to ensure that the athlete maintained the proper position and did not move (An et al., 2019).

#### Jumping Ability

Jumping ability was assessed by squat jump (SJ) [used to assess concentric muscle actions] and countermovement jump (CMJ) [used to assess the slow stretch-shortening cycle (SSC)] through the maximum height reached (cm) through an electronic contact platform (Ergojump; Globus, Codogné, Italy; accuracy: 0.01 m), following the standard procedures (Ramírez-Campillo et al., 2013; Groeber et al., 2020).

#### Taekwondo-Specific High-Intensity Intermittent Efforts

The ability to repeat specific high-intensity intermittent efforts was assessed by the FSKT_MULT_, following previously described protocols (da Silva Santos et al., 2018; da Silva Santos and Franchini, 2018). The performance was determined by the number of kicks in each series, the total number of kicks (total kicks), and the Kick Decrement Index (KDI) during the assessment. To calculate the KDI, the number of kicks applied during the FSKT_MULT_ was considered. The outcomes were calculated using an equation that considers the results of all FSKT series (Eq. 1).


(1) $$\text{KDI}\left(\%\right)=\left[1-\frac{\mathrm{FSKT1}+\mathrm{FSKT2}+\mathrm{FSKT3}+\mathrm{FSKT4}+\mathrm{FSKT5}}{\mathrm{Best}\,\mathrm{FSKT}\times \mathrm{Number}\,\mathrm{of}\,\mathrm{Sets}}\right]\times 100$$

#### Aerobic Fitness

Aerobic fitness was assessed indirectly by the 20-m shuttle run (20MSR) according to the standard procedures (Leger et al., 1988) and previous studies in TKD (Norjali Wazir et al., 2019). The 20MSR outcomes were expressed as total time in minutes from the start to the point of voluntary exhaustion or disqualification.

#### Training Program

Both groups participated in a training program of 12 sessions (4 weeks) with a duration of 90 min per session, which was carried out on 3 non-consecutive days (Monday, Wednesday, and Friday), considering a training load distribution with emphasis on technical development with the permanent intervention of the coach. Previously, both groups were instructed to use the subjective perception of effort scale (RPE 0–10) to control the internal load during the application of the HIIT protocols as a study with similar characteristics (Ouergui et al., 2020). Each training session started with a standardized ∼20 min warm-up consisting of circle jogging and dynamic stretching. Subsequently, all athletes worked in pairs for 50 min (RPE-5). For the first ∼25 min, in pears, each athlete performed 6–8 sets of 12 circular kicking sequences alternately using speed paddles (i.e., kicking with the front leg and then with the back leg, respectively) with ∼2-min passive recovery between sets (RPE-5). Then, during the last ∼25 min, each athlete performed 6–8 sets of 10 free kicks with a 1-min passive recovery, oriented to reaction speed.

After ∼60 min, the training groups (i.e., TS-G and RS-G) were separated from the total group of athletes to execute a HIIT protocol at the same time with the same volume (∼10 min) and distribution [three rounds of 2 min of activity (4 s of repeated efforts with 28 s of active pause; effort/pause ratio of 1:7) with 1 min of passive pause between rounds]. Specifically, TS-G performed HIIT with 4 s of efforts followed by 28 s of pause using alternating circular kicks with both legs at maximum intensity (i.e., all-out), considering an RPE of 10 in front of a partner. This was followed by periods mimicking the guard posture. During passive pause, they hydrated and simulated receiving instructions from the coach and assistants. Meanwhile, the RS-Gs performed 4 s of repeated linear sprints over 5 m followed by 28-s dynamic pauses based on walking. A sound stimulus *via* an iPhone app (Interval Timer, i.e., HIIT Workouts, Deltaworks) connected to a sound system was used to distribute workout time to both training groups.

Finally, both groups concluded the training sessions with a return to calm by performing static stretching exercises for 10 min.

### Procedures

**FIGURE 2:**
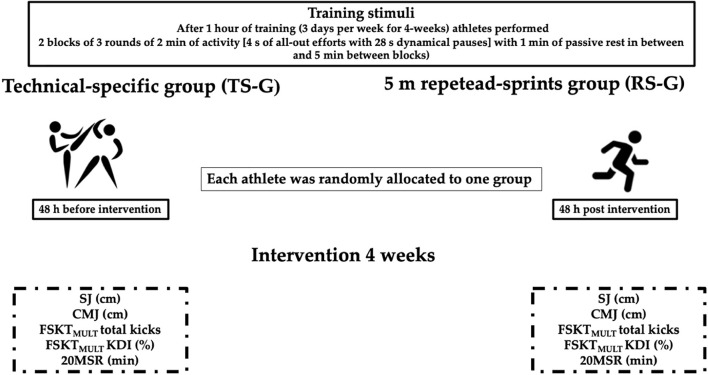
High-intensity interval training with specific techniques (HIIT TS-G) vs. HIIT with repeated sprints (HIIT RS-G) training program. SJ, squat jump; CMJ, countermovement jump; FSKT_MULT_, multiple frequency speed of kick test; KDI, Kick Decrement Index; 20MSR, 20-m shuttle run test.

During the previous week, the athletes completed a familiarization session practicing the corresponding HIIT protocols and physical fitness assessments to reduce the learning effect. The assessments were conducted before and after the application of the training program with 48 h of rest between the first and last training sessions. All assessments were scheduled between 9:00 AM and 11:00 AM and completed in the same order, in the same location (gymnasium with wooden floor), with the same sports clothing, and by the same sports science professional before and after the intervention, previously blinded to the intervention. Previously, all athletes were instructed to: (i) sleep 8 h between each assessment session, (ii) not to modify their usual diet and hydration habits during the days before the assessments, and (iii) not to consume caffeine-containing stimulants. The first session assessed chronological age, height, BM, and fat mass percentage. The second session of assessments considered TKD fitness such as SJ, CMJ, FSKT_MULT_, and 20MSR. A standard warm-up for the sport was performed for 15 min consisting of joint mobility, jogging (5 min), dynamic stretching (3 min), three SJ and CMJ drills (2 min), and low-intensity kicks (5 min). Also, athletes were previously instructed to give their maximum effort during the assessments. The best of two attempts was considered for the performance of all assessments, except for the FSKT_MULT_ and 20MSR maximal assessments. A 2-min rest interval between attempts was implemented, and a 10-min rest interval was applied between each assessment to reduce fatigue effects (for details of design see Figure 2).

### Statistical Analysis

The data analysis was performed with SPSS version 26 for Mac (SPSS Institute, Chicago, IL, United States). The data are presented as mean ± SD. The homoscedasticity of variance and normality was verified by the Levene’s test and the Shapiro–Wilk test, respectively. The relative and absolute reliability of the jumps was verified by the intraclass correlation coefficient (ICC) and the coefficient of variation (CV), respectively, with a 90% CI. The acceptable reliability was determined when an ICC was equal to or greater than 0.80 and CV 10%, respectively (Hopkins, 2000, 2002). The potential bias of the outcomes in both genders and groups was verified using an unpaired and paired *t*-test. The interaction of group (i.e., intersubject factor) TS-G vs. RS-G and time (i.e., intrasubject factor) pre-training vs. post-training was analyzed using repeated-measures mixed ANOVA. If significant effects or interactions were observed, the Bonferroni *post hoc* test was applied to adjust for differences between the means of the two groups. For ANOVA results, effect sizes (ES) were calculated using partial eta-squared (η^2^_p_). Complementarily, the post-intervention changes within and between groups were calculated by Cohen’s *d*, following the classification proposed by Rhea for recreationally trained participants (individuals training consistently for 1–5 years) (trivial <0.25; small 0.25–0.50; moderate 0.50–1.0; large >1.0) (Rhea, 2004). Subsequently, the sample was classified into Rs and NRs using the two technical error (TE) criteria according to a previously established equation (Bonafiglia et al., 2016). NRs were identified and defined as individuals who were unable to demonstrate an increase or decrease (in favor of beneficial changes) in sport-related fitness that was greater than two times the TE away from zero (Ramirez-Campillo et al., 2018). For this study, two replicates of all outcomes analyzed were used to calculate TE. A change beyond two times the TE was a representative of a high probability (i.e., 12 to 1 odds) that the observed response was a true physiological adaptation beyond what might be expected as a result of technical and/or biological variability (Ramirez-Campillo et al., 2018). Therefore, the TEs were as follows: [SJ 2.09 (cm) × 2; CMJ 2.98 (cm) × 2; FSKT_MULT_ total kicks 5.06 (kicks) × 2; FSKT_MULT_ KDI 2.91 (%) × 2; 20MSR 0.87 (min) × 2]. In addition, the Fisher’s exact test was used for comparisons between groups of subjects who were at the 2 × TE calculated on each outcome (NRs) or more than two times the TE (Rs) (Ramirez-Campillo et al., 2018). The level of statistical significance was set at *p* < 0.05.

## Results

### Effect and Interaction of the Factors Analyzed

Table 1 presents the recorded performance of the TKD-related fitness outcomes from before and after the HIIT training program. For pre-training values, statistical differences were found for SJ (*F*_1_,_10_ = 13.96; *p* = 0.04; η^2^*_*p*_* = 0.58) in RS-G (*F*_1,10_ = 5.51; *p* = 0.04; η^2^*_*p*_* = 0.35), and TS-G (*F*_1,10_ = 8.61; *p* = 0.01; η^2^*_*p*_* = 0.46). There was neither a significant effect in the group factor (*F*_1,10_ = 0.22; *p* = 0.64; η^2^*_*p*_* = 0.22) nor a group by time interaction (*F*_1,10_ = 0.17; *p* = 0.68; η^2^*_*p*_* = 0.01).

**TABLE 1:** Effects and response rate of specific techniques (TS-G) vs. repeated sprints (RS-G) on taekwondo-related fitness outcomes (*n* = 12).

	**Pre-training**	**Post-training**	**% change ± DS**	***F*_1,10_; *p*; η** ^2^ ***p***	**ES**	**Rs**	**Pre-training**	**Post-training**	**% change ± DS**	***F*_1,10_; *p*; η** ^2^ ***_*p*_***	**ES**	**Rs**	**ES**
		
**TS-G (*n* = 6)**							**RS-G (*n* = 6)**						**TS-G vs. RS-G**
SJ (cm)	27.1 ± 6.7	29.6 ± 5.0	11 ± 11.2	8.61; 0.01; 0.46*	0.28 (0.02 to 0.54) *Small*	3 (50)	26.1 ± 2.99	28.1 ± 2.79	8 ± 7.03	5.51; 0.04; 0.35*	0.45 (0.06 to 0.85) *Small*	2 (33.3)	−0.21 (−0.63 to 0.21) *Small*
CMJ (cm)	31.0 ± 5.8	30.3 ± 3.5	−1 ± 12.6	0.32; 0.58; 0.03	0.00 (−0.70 to 0.70) *Trivial*	1 (16.6)	27.5 ± 3.89	27.5 ± 4.18	−0 ± 5.3	0.00; 1.0; 0.00	0.06 (−0.35 to 0.47) *Trivial*	0	0.11 (−0.44 to 0.65) *Trivial*
Total Kicks	93.1 ± 9.79	92.3 ± 9.50	−1 ± 10.2	0.07; 0.79; 0.00	−0.06 (−0.81 to 0.68) *Trivial*	1 (16.6)	93.1 ± 9.5	90.6 ± 8.4	− 2.4 ± 5.7	0.67; 0.43; 0.63	−0.18 (−0.66 to 0.31) *Trivial*	0	−0.12 (−0.93 to 0.69) *Trivial*
KDI (%)	6.12 ± 2.51	4.39 ± 1.77	−18 ± 41.8	1.50; 0.24; 0.13	−0.86 (−1.82 to 0.10) *Moderate*	0	3.4 ± 2.4	6.9 ± 4.3	197 ± 328.2	5.96; 0.02; 0.03*	0.38 (−0.11 to 0.87) *Small*	0	0.83 (0.13 to 1.53) *Moderate*
20MSR (min)	7.8 ± 2.6	8 ± 2.9	2 ± 9.9	0.75; 0.79; 0.07	0.10 (−0.03 to 0.23) *Trivial*	0	5.8 ± 2.3	4.4 ± 1.6	11.6 ± 21.1	0.67; 0.43; 0.63	−0.29 (−0.84 to 0.25) *Small*	0	−0.44 (−1.24 to 0.35) *Small*

P, p-value; η^2^*_p_*, partial eta-squared; *p < 0.05, statistical differences from pre-training; % change ± DS, percentage changes in means; ES, effect size with 90% CI; Rs, responders; TS-G, technical specific group; RS-G: repeated-sprint group; SJ, squat jump; CMJ, countermovement jump; KDI, Kick Decrement Index; 20MSR, 20-m shuttle run.

Also, relevantly KDI, the statistical differences were documented for the time factor (*F*_1,10_ = 6.73; *p* = 0.03; η^2^*_*p*_* = 0.40) although only in the RS-G (*F*_1,10_ = 5.96; *p* = 0.03; η^2^*_*p*_* = 0.37) vs. TS-G group (*F*_1,10_ = 1.50; *p* = 0.24; η^2^*_*p*_* = 0.13).

No significant effects and interactions were reported for total kicks and 20MSR in both groups analyzed.

### Magnitude of Change Based on Inference

Relevant findings include an improvement of SJ in both groups: TS-G by 11% (ES = 0.28; small) and RS-G by 8% (ES = 0.45; small) in favor of TS-G (ES = −0.21; small). Similarly, a decrease in KDI was documented in TS-G by −18% (ES = −0.86; moderate), while an increase of 197% was documented in RS-G (ES = 0.38; small) with moderate differences (ES = 0.83) in favor of TS-G. Also, an increase in 20MSR performance of 2% was documented in TS-G (ES = 0.10; trivial), while a decrease of 11.6% (ES = −0.29; trivial) was documented in RS-G with a small difference in favor of TS-G (ES = −0.44).

For CMJ height, performance decreases in TS-G of −1% (ES = 0.00; trivial) were documented, while the maintenance of performance in RS-G with a trivial decreased difference (ES = 0.11; trivial) was in favor of RS-G.

### Inter-Individual Variability Responses to the High-Intensity Interval Training Program

**FIGURE 3:**
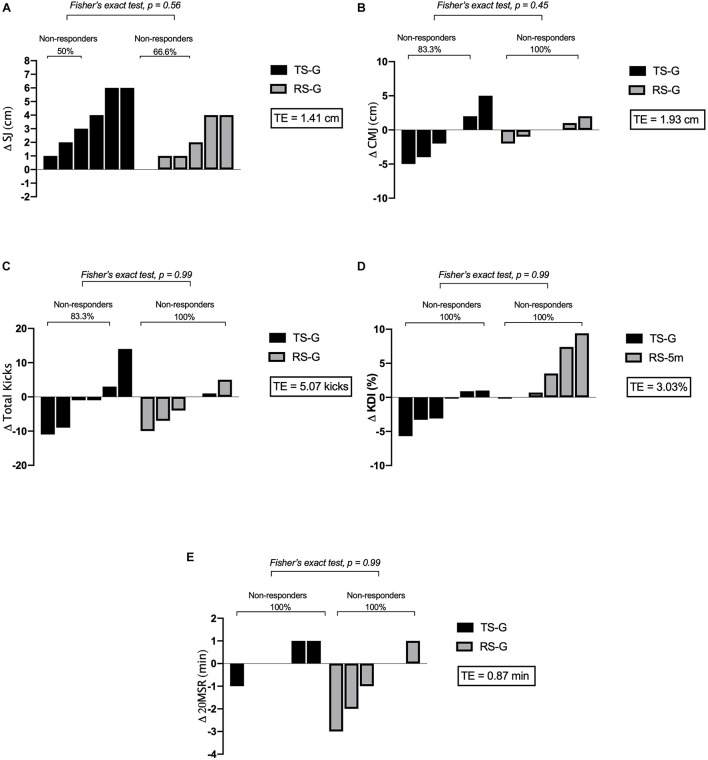
Inter-individual variability of the response of both training groups on taekwondo-related fitness. **(A)** SJ, **(B)** CMJ, **(C)** FSKT_MULT_ total kicks, **(D)** KDI, and **(E)** 20MSR. Δ, post-pre changes; TS-G, technical-specific group; RS-G, repeated-sprint group; TE, technical error.

Table 1 and Figure 3 illustrate in detail the Rs athletes of both groups after the HIIT training program. Overall, Rs were documented for the TS-G in SJ, CMJ, and total kicks. In RS-G, Rs were documented for SJ. NRs athletes were documented in all outcomes in both training groups.

## Discussion

This study investigated the effect of 4 weeks of HIIT with specific techniques vs. repeated-sprints and analyzed the inter-individual variability (classified 538 into Rs and NRs) on sport-related fitness in TKD athletes. Among the main findings, there were significant increases for SJ in both groups (i.e., TS-G and RS-G). In addition, in TS-G group, they were informed of moderate decreases in KDI and percentage increases in 20MSR performance. In addition, athlete Rs were observed in both groups, although with a higher trend in TS-G. In contrast, a significant decrease in KDI performance was reported in RS-G. Therefore, the hypothesis was not fulfilled, i.e., TS-G was not comparatively superior to RS-G in sport-related fitness in TKD athletes, nor was a significantly higher proportion of athlete Rs documented in TS-G vs. RS-G.

### Jumping Ability

Concerning jumping ability after the HIIT program, these findings add to the inconsistent evidence. In this regard, for example, recently, Ojeda-Aravena et al. (2021) reported the absence of significant increases in SJ performance and CMJ height after applying HIIT with specific techniques [three rounds of 2 min of interspersed repeated kicks (4-s: 28-s) with a 1-min rest in between] added to regular training after 4 weeks (Ojeda-Aravena et al., 2021). Furthermore, Ouergui et al. (2021) recently found no significant increases in CMJ height performance after comparing the addition of two sessions to regular HIIT training (two blocks of combat-based training separated by 4 min) in different size areas following 8 weeks (Ouergui et al., 2021). In contrast, significant increases in CMJ height performance (*p* = 0.01) were reported after 4 weeks of HIIT with specific techniques (three sets of 10 repetitions of 6 s of repeated kicks with 10 s of passive rest between repetitions and 3 min of passive rest between sets) vs. a HIIT based on repeated-sprints although not vs. the control group (Ouergui et al., 2020). In addition, other reports document significant increases in CMJ height (*p* < 0.001) after applying HIIT based on repeated-sprints (6–8 sets at 80–100% of HRmax with 30 s of active pauses between sets) with different effort/pause ratios vs. a usual training or control group for 4 weeks (Seo et al., 2019). Specifically, the authors reported that the HIIT 1:4 s group or the HIIT 30:120 s group documented a greater increase in height jump performance (Seo et al., 2019).

However, the heterogeneity of the HIIT protocols applied in this sport does not allow us to be conclusive about adaptations in jumping ability. In this regard, for example, the HIIT studies in TKD, which were analyzed, use different configurations of effort/pause ratio. Therefore, the increased neuromuscular stress involved during high-intensity activity could affect the adaptation of lower-body dynamic strength in response to training (Seo et al., 2019). Furthermore, the different motor patterns used during HIIT (running, jumping, and specific techniques) involve different lower extremity neuromuscular stresses and potentially different adaptations. In fact, HIIT based on repeated-sprints shows muscular adaptations (e.g., neuromuscular, metabolic, and physical performance) both in TKD athletes (Monks et al., 2017; Seo et al., 2019) and in endurance athletes and collective sports (Kohn et al., 2011; Kinnunen et al., 2019). In this sense, in light of the results, HIIT with specific techniques could demonstrate relevant effects on performance in the ability to generate vertical dynamic force through jumping capacity, for which further research is required.

In turn, most of the HIIT studies in TKD, which were analyzed, apply HIIT in independent sessions, so that an increase in training volume could increase the probability of large increases in jumping capacity. In this sense, the lack of reports in SJ does not yet allow us to conclude on the potential benefits that HIIT could have on the dynamic muscular strength of the lower limbs. In this regard, reports mostly report the use of CMJ for the assessment of the slow stretch shortening cycle during vertical jumping. Adding to the abovementioned reports, it is likely that the lack of movement specificity using the SSC may have influenced the findings of this study, even taking into account the considerable strain and neuromuscular load promoted by the different HIIT formats (Laursen and Buchheit, 2018; Ouergui et al., 2020). Furthermore, it could be inferred that the lack of training volume may have influenced the absence of significant increases in the performance of both jumps analyzed in this study.

### Taekwondo-Specific High-Intensity Intermittent Efforts

The analysis of the findings of this study on this TKD-specific ability is similar to a recent report in TKD that applied a HIIT with specific techniques (three blocks of six sets of 10 s of repeated kicks, with a passive rest of 10 s between sets with 1-min rest between blocks) in addition to the usual training vs. the control group after 4 weeks of training (Aravena et al., 2020). The authors reported significant increases in total kicks (*p* < 0.001) but no significant decreases in KDI (Aravena et al., 2020). In contrast, the authors Ojeda-Aravena et al. (2021) reported no significant changes in FSKT_MULT_ outcomes although they did report a positive percentage and ES in both groups (HIIT vs. control) in total kicks and KDI (Ojeda-Aravena et al., 2021). Overall, these results support the notion, that is, the application of specific training stimuli (i.e., TS-G) in combat sports, which could positively influence performance when using HIIT protocols (Franchini, 2020). However, at present, the evidence is still inconclusive, although it appears that increasing the magnitude components of the training load (i.e., volume, intensity, and decreasing training density), as well as the type of exercises used, could positively influence significant increases in this sport-specific capacity.

### Aerobic Fitness

Regarding the findings obtained after the HIIT program on aerobic fitness performance, the results of this study are consistent with the study by Ojeda-Aravena et al. (2021) who document the increases of ∼9 to 12% in both technique-specific HIIT training and habitual training in TKD athletes after 4 weeks (Ojeda-Aravena et al., 2021). In contrast, these results differ from other studies with HIIT in TKD, in particular, using repeated-sprints and specific techniques reporting significant increases in 20MSR performance, significant increases in VO_2_max (assessed on the treadmill), and TKD-specific progressive aerobic assessment (Monks et al., 2017; Seo et al., 2019; Ouergui et al., 2020, 2021). In this sense, HIIT is an effective training method to optimize cardiorespiratory fitness such as athletes in combat sports (Billat, 2001; Laursen and Buchheit, 2018; Franchini et al., 2019; Vasconcelos et al., 2020). The absence of positive changes would be related to the lack of time associated with high-intensity activity, considering that to increase cardiorespiratory fitness, the total cumulative time of high-intensity work should be greater than 10 min (Laursen and Buchheit, 2018). In addition, a greater number of rounds will probably be required to increase the time of the HIIT protocol to simulate a TKD competition.

### Inter-Individual Variability Responses to High-Intensity Interval Training Program

Another aim of this study was to analyze the inter-individual variability of the athletes after the HIIT program. In this sense, these results are similar to those reported recently using a similar design to this study documented Rs for SJ (*n* = 2), total kicks (*n* = 1), and KDI (*n* = 2) (Ojeda-Aravena et al., 2021). Evidence has also been previously reported on jumping ability after 7 weeks of training with a significant increase in the number of Rs of athletes in the plyometric training group vs. habitual training (Ramirez-Campillo et al., 2018). Regarding high-intensity outcomes, recently comparing three HIIT protocols (work/rest ratio = 1:8) with different work configurations (30:240 s; 15:120 s; 5:40 s vs. control group) for 4 weeks at a significantly higher proportion of Rs at maximal speed in the 30-s sprint test vs. control, specifically in the 30:240 s work interval in healthy individuals (Schulhauser et al., 2020). In contrast, about cardiorespiratory fitness results, most HIIT studies that are analyzing the inter-individual response document significant increases in VO_2_max and a higher proportion of Rs in endurance athletes (Bonafiglia et al., 2016), cyclists, triathletes (Bratland-Sanda et al., 2020), and recreationally active individuals (Gurd et al., 2016; Schulhauser et al., 2020). Accordingly, the inter-individual variability of observed responses to training, such as HIIT, according to the study by Walsh et al. (2020), is a combination of: (i) individual responses to perseverative exercise training (subject-training interaction), (ii) day-to-day biological variation and TE (random variation), and (iii) physiological responses associated with behavioral/maturational changes, not attributable to exercise (e.g., within-person variability) (Walsh et al., 2020). This includes genetic (Mann et al., 2014; Sparks, 2017; Bonafiglia et al., 2020; Del Coso et al., 2020), climatic (Corbett et al., 2018), cognitive (Atkinson and Batterham, 2015), stress and sleep status (Mann et al., 2014), gender, age, time of day variation (Mann et al., 2014; Sparks, 2017), training status (Pickering and Kiely, 2019), physiological (Williamson et al., 2017; Atkinson et al., 2019), and statistical outcomes (Swinton et al., 2018; Bonafiglia et al., 2020; Chrzanowski-Smith et al., 2020).

### Limitations

Possible limitations of this study include: (i) the training status achieved by the athletes during the year; (ii) the limited number of athletes analyzed; (iii) the short duration of the program, given the level of the athletes; (iv) the lack of the control group; (v) the lack of the magnitude of the load applied.

### Highlights and Practical Applications

Although requiring further study, the technique-specific HIIT protocols using the temporal structure of combat could be an alternative to incorporate as part of the training session during inter-competitive periods (e.g., during a shock microcycle) due to the limited time available to athletes to cope with the demands of this period. In addition, these HIIT protocols can be executed in reduced places. In turn, coaches could use the inter-individual response analysis as a practical monitoring tool to follow the training progress of each athlete.

## Conclusion

The addition to the regular training of a HIIT with TS-G and RS-G associated with intervals and similar structure of the combat during 4 weeks of training can improve the concentric characteristics of lower limb performance, although they were not the sufficient stimuli in the other components of TKD-related fitness.

## Data Availability Statement

The raw data supporting the conclusions of this article will be made available by the authors, without undue reservation.

## Ethics Statement

The studies involving human participants were reviewed and approved by the Universidad Autónoma de Chile (080-18). Written informed consent to participate in this study was provided by the participants’ legal guardian/next of kin.

## Author Contributions

AO-A and TH-V contributed to the conception and performed the data interpretation or analysis. TH-V, PV-B, JC-L, JZ-B, and JG-G performed the implementation of the study. AO-A, PV-B, TH-V, JC-L, JZ-B, and JG-G contributed to the manuscript preparation, proofreading of important intellectual content, and supervision. All authors have read and agreed to the published version of the manuscript.

## Conflict of Interest

The authors declare that the research was conducted in the absence of any commercial or financial relationships that could be construed as a potential conflict of interest.

## Publisher’s Note

All claims expressed in this article are solely those of the authors and do not necessarily represent those of their affiliated organizations, or those of the publisher, the editors and the reviewers. Any product that may be evaluated in this article, or claim that may be made by its manufacturer, is not guaranteed or endorsed by the publisher.
